# Regulation of transplanted mesenchymal stem cells by the lung progenitor niche in rats with chronic obstructive pulmonary disease

**DOI:** 10.1186/1465-9921-15-33

**Published:** 2014-03-25

**Authors:** Wan-Guang Zhang, Li He, Xue-Mei Shi, Si-Si Wu, Bo Zhang, Li Mei, Yong-Jian Xu, Zhen-Xiang Zhang, Jian-Ping Zhao, Hui-Lan Zhang

**Affiliations:** 1 Department of Surgery, Tongji Hospital of Tongji Medical College, Huazhong University of Science and Technology, Wuhan, Hubei, China; 2 Department of Respiratory Medicine, Tongji Hospital of Tongji Medical College, Huazhong University of Science and Technology, 1095, Jie Fang Road, Han Kou District, Wuhan, Hubei 430030, China; 3 Department of Respiratory Medicine, Jingzhou Central Hospital, Jingzhou, Hubei, China

**Keywords:** Chronic obstructive pulmonary disease, Mesenchymal stem cells, Alveolar epithelial type II cells, Niche

## Abstract

**Background:**

Stem cell transplantation is a promising method for the treatment of chronic obstructive pulmonary disease (COPD), and mesenchymal stem cells (MSCs) have clinical potential for lung repair/regeneration. However, the rates of engraftment and differentiation are generally low following MSC therapy for lung injury. In previous studies, we constructed a pulmonary surfactant-associated protein A (SPA) suicide gene system, rAAV-SPA-TK, which induced apoptosis in alveolar epithelial type II (AT II) cells and vacated the AT II cell niche. We hypothesized that this system would increase the rates of MSC engraftment and repair in COPD rats.

**Methods:**

The MSC engraftment rate and morphometric changes in lung tissue *in vivo* were investigated by *in situ* hybridization, hematoxylin and eosin staining, Masson’s trichrome staining, immunohistochemistry, and real-time PCR. The expression of hypoxia inducible factor (HIF-1α) and stromal cell-derived factor-1 (SDF-1), and relationship between HIF-1α and SDF-1 in a hypoxic cell model were analyzed by real-time PCR, western blotting, and enzyme-linked immunosorbent assay.

**Results:**

rAAV-SPA-TK transfection increased the recruitment of MSCs but induced pulmonary fibrosis in COPD rats. HIF-1α and SDF-1 expression were enhanced after rAAV-SPA-TK transfection. Hypoxia increased the expression of HIF-1α and SDF-1 in the hypoxic cell model, and SDF-1 expression was augmented by HIF-1α under hypoxic conditions.

**Conclusions:**

Vacant AT II cell niches increase the homing and recruitment of MSCs to the lung in COPD rats. MSCs play an important role in lung repair and promote collagen fiber deposition after induction of secondary damage in AT II cells by rAAV-SPA-TK, which involves HIF-1α and SDF-1 signaling.

## Background

The pathogenesis of chronic obstructive pulmonary disease (COPD) is characterized by upregulation of inflammatory processes that lead to irreversible events such as apoptosis of epithelial cells and proteolysis of the terminal air space and lung extracellular matrix components [1]. Recent studies have revealed that mesenchymal stem cells (MSCs) contribute to lung tissue regeneration and protection [2-6]. However, the rates of engraftment and differentiation are generally low following MSC therapy for lung injury. Therefore, there is a need for new methods to improve the rates of engraftment and differentiation.

We have previously established a pulmonary surfactant-associated protein A (SPA) suicide gene system using adeno-associated virus-SPA-thymidine kinase (rAAV-SPA-TK) [7], which induces apoptosis in alveolar epithelial type II (AT II) cells and vacates the AT II cell niche. We hypothesized that the SPA suicide gene system would increase the rates of MSC engraftment and repair in COPD rats. Interestingly, the rate of MSC engraftment increased significantly in COPD rats, which was accompanied by abnormal pulmonary fibrosis, suggesting that the MSCs did not undergo the expected differentiation into epithelial cells. We observed increased expression of hypoxia inducible factor (HIF-1α) and stromal cell-derived factor-1 (SDF-1) and established a hypoxic cell model to explore the mechanisms underlying the recruitment and abnormal differentiation of MSCs in the AT II cell niche.

## Methods

### Animals and model

Fifty Sprague Dawley (SD) female rats (200–250 g) were procured from the Center of Experimental Animals, Tongji Medical College, Huazhong University of Science and Technology (Wuhan, China). All experiments were approved by the Institutional Animal Care and Use Committee of Tongji Medical College, Huazhong University of Science and Technology. Rats were given free access to water and a standard rodent diet (no. 2920, Harlan Laboratories, Indianapolis, IN, USA). To establish a rat COPD model, rats were given 0.2 ml of 1 mg/ml lipopolysaccharide (LPS, Sigma) by tracheal instillation on days 1 and 14, and passive cigarette smoking (20 filtered commercial cigarettes each) for 1 h twice per day for 2 months continuously except for days 1 and 14 [8]. Oxygen levels were monitored using a digital oxygen meter (CYES-1, Shanghai JiaDingXueLian Factory, Shanghai, China) during passive cigarette smoking. The oxygen content was as low as 17.1% when rats were exposed to cigarette smoke in a Perspex cage. Control animals were housed in cages and inhaled clean room air only in the absence of LPS administration.

### Isolation and expansion of rat bone marrow-derived MSCs

Rat MSCs were isolated by flushing the cells from femurs and tibias of 6-week-old male SD rats (Center of Experimental Animals, Tongji Medical College) with Dulbecco’s modified Eagle’s medium (DMEM, Hyclone, Logan, UT, USA) containing 1% penicillin/streptomycin. The cell suspension was applied to a Percoll gradient (Pharmacia, Uppsala, Sweden) and centrifuged for 30 min. The mononuclear cells were collected and resuspended in DMEM containing 10% fetal bovine serum (Gibco, Rockville, MD, USA). The cells were plated at a density of 1 × 10^6^ cells/cm^2^ and cultured at 37°C in a 5% CO_2_ incubator. After 24 h, the cultures were washed with PBS to remove non-adherent cells, and the remaining adherent cells were cultured in fresh medium until confluency. The medium was changed every 3–4 days. For flow cytometric analysis, passage 2 MSCs were stained with antibodies against CD11b, CD45, CD29, and CD105 (BD Pharmingen, San Diego, CA, USA). The cells were also subjected to osteogenic, adipogenic, and chondrogenic differentiation assays [9].

### Experimental design

Rats were randomly divided into five groups: A) normal control group; B) COPD group; C) COPD + ^60^CO γ irradiation + MSCs transplantation group; D) COPD + rAAV-SPA-TK injection + ^60^CO γ irradiation + MSC transplantation group; E) COPD + AAV injection + ^60^CO γ irradiation + MSC transplantation group. COPD rats were injected with approximately 3 × 10^11^ v.g. rAAV-SPA-TK *via* the tail vein on day 61. The control COPD + AAV injection + ^60^CO γ irradiation + MSC transplantation group was intraperitoneally (i.p.) injected with AAV. Next, approximately 100 mg/kg ganciclovir was i.p. injected for 20 days from day 62. The rats underwent whole body exposure to ^60^CO γ irradiation of 7.5 Gy once on day 90. Within 4 h of irradiation, approximately 4 × 10^6^ MSCs isolated from male rats were delivered systemically into female rats in approximately 200 μl sterile saline *via* the tail vein as described previously [10]. The rats were sacrificed on the day 121. A left lung lavage was performed for each rat. Transplanted MSCs were detected by Y chromosome fluorescent *in situ* hybridization. The right lung tissues were sampled for morphometric analysis and immunohistochemical staining.

In our previous study, we observed AT II cell apoptosis *in vitro*[7] and *in vivo*. Therefore, we determined whether the rAAV-SPA-TK system could induce apoptosis of AT II cells and vacate their niche. The rAAV-SPA-TK system uses rAAV for targeted killing under the control of the promoter of the surfactant protein A gene (expressed by AT II cells). In the *in vivo* experiment, rats were randomly divided into four groups: 1) normal control; 2) COPD; 3) COPD + rAAV-SPA-TK injection; 4) COPD + AAV injection. COPD rats were injected with approximately 3 × 10^11^ v.g. rAAV-SPA-TK/AAV *via* the tail vein on day 61. Next, approximately 100 mg/kg ganciclovir was i.p. injected for 20 days from day 62. The rats were sacrificed on day 90. TUNEL assays were then performed (Additional file 1: Figure S1). The results showed that the rAAV-SPA-TK system (the recombinant rAAV-SPA-TK gene was indeed encapsidated in the AAV capsid structure) also increased AT II cell apoptosis induced by ganciclovir *in vivo* and vacated AT II cell niches.

### TUNEL assay for apoptosis detection

Paraffin-embedded samples were cut to a thickness of 4–5 μm, rehydrated, and then incubated with protease K solution for 30 min at room temperature (RT). After two washes with PBS, the samples were incubated with TUNEL reaction solution (Boster, Wuhan, China) at 37°C for 60 min. The transforming solution was then added followed by incubation at 37°C for 30 min. Staining was developed with diaminobenzidine tetrahydrochloride for 10 min. Then, the samples were counterstained with hematoxylin for 10 min, dehydrated in graded alcohol, and covered with resin. The criterion for positive staining was pale brown-stained nuclei.

### Y chromosome fluorescence *in situ* hybridization

Y chromosome fluorescence *in situ* hybridization for gender mismatch transplantation between male donors and female recipients has been described using FITC-labeled DNA probes specific for the rat Y chromosome (Cambio, Cambridge, UK) [11]. Frozen lung sections were warmed to RT and then dried for 3 h. The dried sections were washed twice in DEPC-PBS at RT for 5 min each and then fixed in 4% paraformaldehyde in DEPC-PBS at RT for 20 min. After serial dehydration in ethanol, the samples were placed on a hot plate and Y chromosome probes were added to the sections. Tissue sections and probes were denatured at 85°C for 5 min and then incubated at 4°C for 10 min before overnight incubation at 37°C. On the second day, the coverslips were carefully removed and the sections were washed with 2× SSC at 60–65°C for 15 min, 2× SSC at RT for 5 min, and finally 0.1× SSC for 10 min. Then, the sections were sequentially incubated with buffer I for 5 min, buffer II for 15 min, and anti-digoxigenin-alkaline phosphatase complex for 2 h. After washing twice with both buffer I and II, the sections were incubated in NBT/BCIP for 10 min and then counterstained with hematoxylin.

### Morphometric analysis and immunohistochemical staining

One set of lung paraffin sections were cut at 7.5 μm, deparaffinized, rehydrated, and then stained with hematoxylin and eosin (HE) or Masson’s trichrome stain (Masson stain) for morphometry or collagen fiber detection, respectively. Morphological alterations were observed in the lung. Mean linear intercept (MLI), mean alveolar number (MAN), and pulmonary alveolar area (PAA) were measured using a HPIAS-100 automatic image analyzer [12,13] in at least eight fields for each rat to obtain the mean values. Collagen area on the basal membrane of airway was analyzed in 3-5 bronchioles with basement membrane perimeter (Pbm) <1000 μm on each slide. The result was expressed as collagen staining area of per micrometer length of basement membrane of bronchioles.

Another set of paraffin-embedded sections were microwaved for 20 min in 10 mM citrate buffer (pH 6.0) for antigen retrieval and permeabilized in 0.1% Triton X-100/PBS (PBS-T) for 3 × 15 min. After blocking for 1 h in 10% normal donkey serum/PBS-T, the sections were incubated at 4°C overnight with an anti-HIF-1α antibody (Novus Biologicals, Littleton, CO, USA), anti-SDF-1 antibody (R&D), or nonspecific isotype IgG as a negative control. After washing, the sections were incubated for 2 h with secondary antibodies, and the nuclei were counterstained with TOPRO-3 (Invitrogen, Carlsbad, CA, USA). The sections were mounted with 30% glycerol in PBS and visualized by laser confocal microscopy.

### Measurement of bronchoalveolar lavage fluid (BALF) cytokines by enzyme-linked immunosorbent assay (ELISA)

BALF cytokines levels were determined by commercial ELISA kits in accordance with the manufacturer’s instructions. ELISA kits for detection of interleukin (IL)-6, IL-8, and tumor necrosis factor-α (TNF-α) in BALF were obtained from Boster (Wuhan, China) with detectable concentration ranges of 15.6–1000 pg/ml, 62.5–4000 pg/ml, and 15.6–1000 pg/ml, respectively.

### Hypoxic cell model

To investigate the effect of hypoxia on the relationship between SDF-1 and HIF-1α, A549 cells were cultured under hypoxic (1.5% O_2_) or normoxic conditions for 48 h. Then, siHIF-1α and scrambled negative control siRNAs were transfected into the cells with Lipofectamine™ 2000 for 48 h under hypoxic conditions (see Table 1 for siRNA sequences). The levels of HIF-1α and SDF-1 mRNAs were measured by real-time PCR SDF-1 protein levels in the culture medium were measured using a commercial ELISA kit (R&D Systems, Minneapolis, MN, USA). Western blotting was used to detect the expression of HIF-1α protein in the cells.

**Table 1:** siRNA sequences

**Gene name**	**siRNA sequence**	
HIF-1α	Forward	5′-CUGAUGACCAGCAACUUGAdTdT-3′
	Reverse	5′-UCAAGUUGCUGGUCAUC AGdTdT-3′
Negative control	Forward	5′-AGUUCAACGACC AGUAGUCdTdT-3
	Reverse	5′ -GACUACUGGUCGUUG AdTdT-3′

### Western blotting

Lung tissues or A549 cells were harvested on ice in radioimmunoprecipitation assay buffer (50 mmol/l Tris–HCl, pH 7.4, 1% NP-40, 0.25% Na-deoxycholate, 150 mmol/l NaCl, 1 mmol/l EDTA, 1 mmol/l phenylmethylsulfonyl fluoride, 1 mg/ml aprotinin, 1 mg/ml leupeptin, 1 mg/ml pepstatin, 1 mmol/l sodium orthovanadate, and 1 mmol/l sodium fluoride) and then centrifuged at 10,000 *g* for 15 min at 4°C. Protein concentrations were determined using the BCA protein assay (Pierce) with bovine serum albumin as the standard. Samples were boiled at 100°C for 10 min in sample buffer. Equal amounts of protein (100 μg per sample) were separated by electrophoresis on 7.5–12% Tris-glycine sodium dodecyl sulfate polyacrylamide gels. After electrophoresis, the proteins were transferred onto nitrocellulose membranes that were then blocked and incubated with anti-HIF-1α (1:500), anti-SDF-1 (1:500, Novus Biologicals), and anti-β-actin (1:2000, Sigma-Aldrich) antibodies at 4°C overnight. After washing, the membranes were incubated with secondary antibodies and then reacted with ECL chemiluminescent horseradish peroxidase detection reagents (Amersham Biosciences, Piscataway, NJ, USA). The blots were scanned and analyzed on a Storm 860 PhosphorImager (GE Healthcare, Fairfield, CT, USA). To quantify the protein of interest, protein abundance was normalized relative to the loading control (β-actin) by densitometry.

### Real-time PCR

Total RNA was extracted from frozen tissue samples using an RNeasy Mini kit (Qiagen, Valencia, CA, USA). The RNA concentrations of the samples were determined using a NanoDrop 1000 (NanoDrop, Wilmington, DE, USA). Reverse transcription was performed with 1 μg RNA, random hexamers, and oligo (dT) 12–18 using a SuperScript III First-Strand Synthesis-Super Mix kit (Invitrogen). mRNA levels were quantified by real-time PCR using SYBR Green I dye. The primers (5′ to 3′) were as follows. Human HIF-1α (GenBank: NM_001530.3): forward, AGTGTACCCTAACTAGCCGAGGAA; reverse, CTGAGGTTGGTTACTGTTGGTATC; amplicon size, 113 bp. Rat HIF-1α (GenBank: NM_024359.1): forward, CGCAGTGTGGCTACAAGAAA; reverse, TATCGAGGCTGTGTCGACTG; amplicon size, 125 bp. Human SDF1 (GenBank: NM_000609.5): forward, GAGCCAACGTCAAGCATCTCAA; reverse, TTTAGCTTCGGGTCAATGCACA; amplicon size, 109 bp. Rat SDF1 (GenBank: NM_022177.3): forward, TTCCGCTTCTCACCTCTGTA; reverse, TGGTTAATTCTAGGCATGTTCTC; amplicon size, 193 bp. Human β-actin (GenBank: NM_001101.3): forward: GCAAGCAGGAGTATGACGAG; reverse, CAAATAAAGCCATGCCAATC; amplicon size, 144 bp. Rat β-actin (GenBank: NM_031144.3): forward, CTAAGGCCAACCGTGAAAAGA; reverse, CCAGAGGCATACAGGGACAAC; amplicon size, 103 bp. Assays were performed in triplicate on an ABI 7900HT Fast Real Time PCR system (Applied Biosystems, Foster City, CA, USA). PCR conditions were 10 min at 95°C followed by 40 cycles of 30 s at 95°C, 60 s at 60°C, and then 15 s at 65°C. Data were normalized to β-actin mRNA levels (∆∆CT analysis) as indicated.

### Statistical analysis

Values are expressed as the means ± SD. Intergroup differences were assessed using the Student’s paired *t*-test. Analysis of variance was used to assess the differences between multiple groups. A value of *P* < 0.05 was considered statistically significant. All evaluations were performed by investigators blinded to the experimental groups.

## Results

### rAAV-SPA-TK + MSCs intervention decreases the apoptosis of alveolar epithelial cells

**Figure 1:**
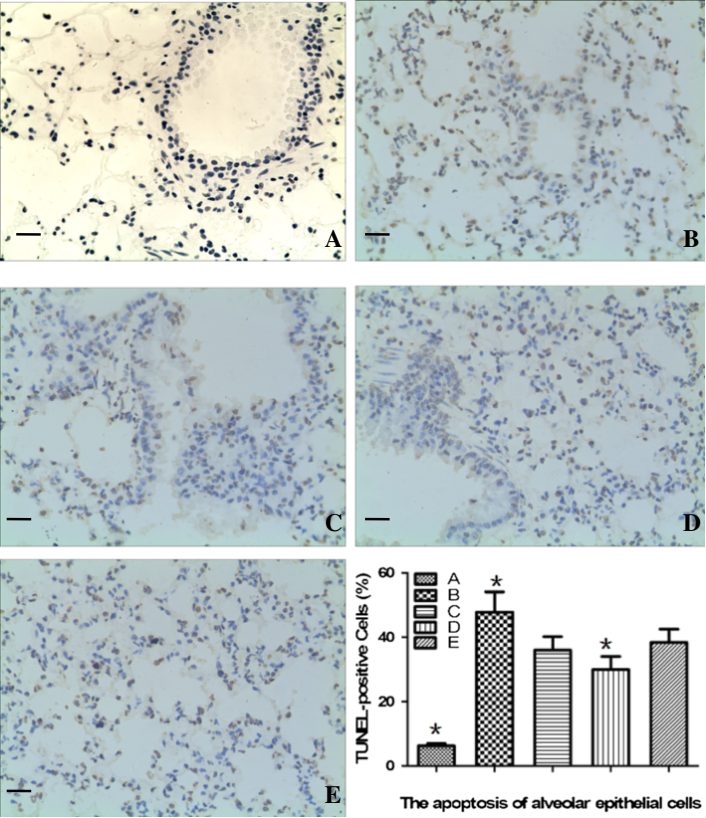
**Apoptosis of alveolar epithelial cells.** TUNEL assays were performed on the rat lung. Nuclei were stained yellow in apoptotic lung cells. The numbers of these cells were significantly increased in groups **B**, **C**, **D**, and **E** compared with those in group **A**. The lowest number of apoptotic cells was in group **D**. The percentage of TUNEL-positive cells was calculated by the ratio of TUNEL-positive cells to the total cell number in 10 fields at 400× magnification from each section. Scale bars = 100 μm. *P < 0.01 compared with the other four groups.

Nuclei were stained yellow in apoptotic alveolar epithelial cells (Figure 1). The numbers of these cells were increased in groups B, C, D, and E. The least number of apoptotic cells was present in group D. The decrease in apoptotic lung cells may because of the transplantation of MSCs.

### rAAV-SPA-TK intervention increases the recruitment of MSCs but induces pulmonary fibrosis

**Figure 2:**
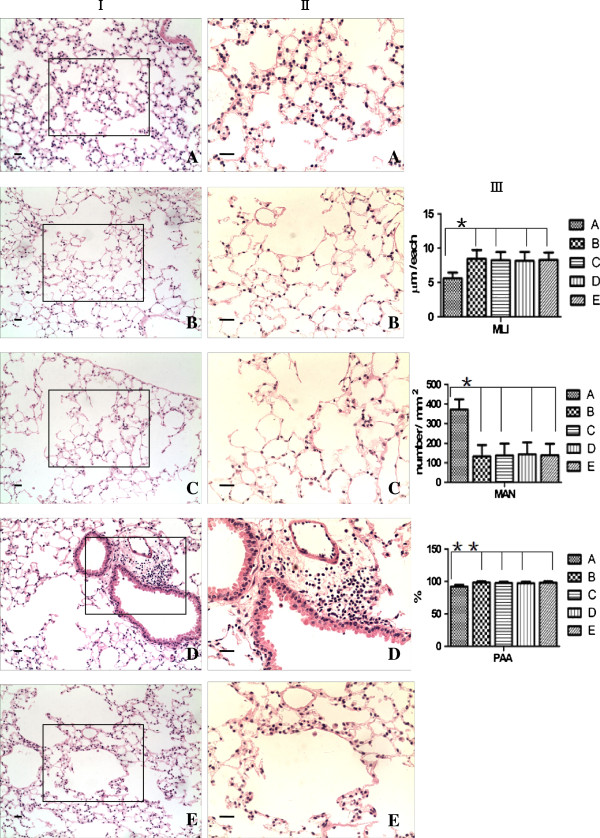
**Morphometric analysis of HE-stained lung tissue.** (**I**, ×100; **II**, ×200). **A)** normal control group; **B)** COPD group; **C)** COPD + ^60^CO γ irradiation + MSCs transplantation group; **D)** COPD + rAAV-SPA-TK injection + ^60^CO γ irradiation + MSC transplantation group; **E)** COPD + AAV injection + ^60^CO γ irradiation + MSC transplantation group. In group **B**, there was a decreased alveolar number (MAN), enlarged mean linear intercept (MLI), and increased pulmonary alveolar area (PAA). There were no statistical differences among groups **B**, **C**, **D** and **E** for MAN, MLI, and PAA. However, in group **D**, there was high cell infiltration. Scale bars = 100 μm. The results are expressed as the mean ± SD, n = 10 in each group. *P < 0.01, **P < 0.05.

**Figure 3:**
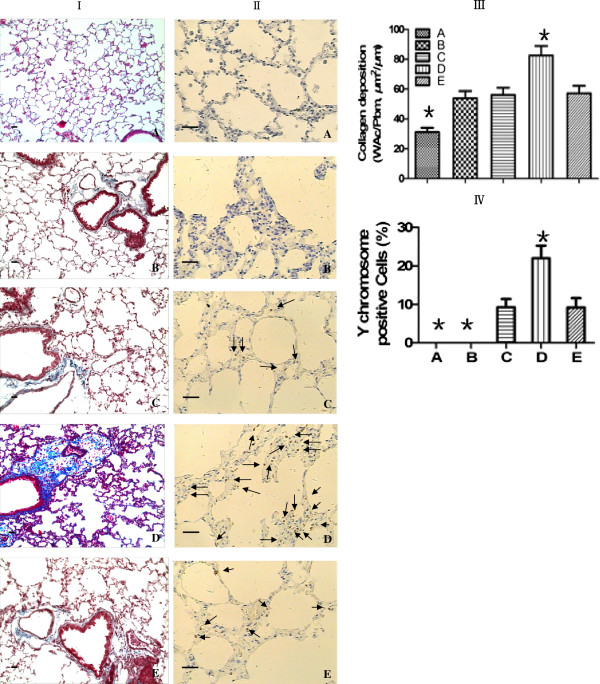
**The increase of MSC homing is associated with collagen fiber deposition. A)** normal control group; **B)** COPD group; **C)** COPD + ^60^CO γ irradiation + MSCs transplantation group; **D)** COPD + rAAV-SPA-TK injection + ^60^CO γ irradiation + MSC transplantation group; **E)** COPD + AAV injection + ^60^CO γ irradiation + MSC transplantation group. **(I)** Morphological appearance of lung sections stained with Masson’s trichrome (×100) showed hardly any collagen deposition in the normal control group **(A)**. Compared with COPD **(B)**, COPD + ^60^CO γ irradiation + MSCs transplantation **(C)**, and COPD + AAV injection + ^60^CO γ irradiation + MSC transplantation **(E)** groups, there was an obvious increase in collagen deposition around vessels and bronchia in the COPD + rAAV-SPA-TK injection + ^60^CO γ irradiation + MSC transplantation group **(D)**. Furthermore, blue collagen fibers were found in lung interstitial spaces. **(III)** The result was expressed as Wac/Pbm (μm^2^/μm) (collagen area on the basal membrane of airway/the perimeter of the basement membrane of bronchioles.**(II, IV)** Detection of Y chromosome-specific signals by fluorescent *in situ* hybridization (FISH) (×200). The nuclei were stained yellow in cells with a Y chromosome (indicated by arrows). These cells were found in groups **C**, **D** and **E**, but more cells were present in group **D**. The percentage of Y- chromosome positive cells was calculated by the ratio of Y- chromosome positive cells to the total cell number in 5-10 fields at 400× magnification from each section. Increased numbers of MSCs after rAAV-SPA-TK injection suggested that stem cell niches increased the homing of MSCs to the lung. Scale bars = 100 μm. *P < 0.01 compared with the other four groups.

Female SD rats in the COPD group showed severe alveolar destruction compared with that in the normal control group with respect to MLI, MAN, and PAA. Pathological changes in MLL, MAN, and PAA were improved in all MSC transplantation groups compared with those in the COPD group, but the data were not statistically significant (Figure 2). In the COPD + rAAV-SPA-TK injection + ^60^CO γ irradiation + MSC transplantation group, collagen fibers increased significantly around bronchi and vessels, and light blue collagen fibers were found in the interstitial lung (Figure 3I, III). Cells with yellow-stained nuclei were found in MSC transplantation groups, indicating that the quantity of cells with a Y chromosome was the highest in the group injected with rAAV-SPA-TK (Figure 3II, IV). No cells with a Y chromosome were found in normal control or COPD groups (these two groups did not receive transplanted MSCs). These data suggest that injection with rAAV-SPA-TK increases the recruitment of MSCs, but also induces pulmonary fibrosis.

### Enhanced immunohistochemical staining of HIF-1α and SDF-1 following MSC transplantation

**Figure 4:**
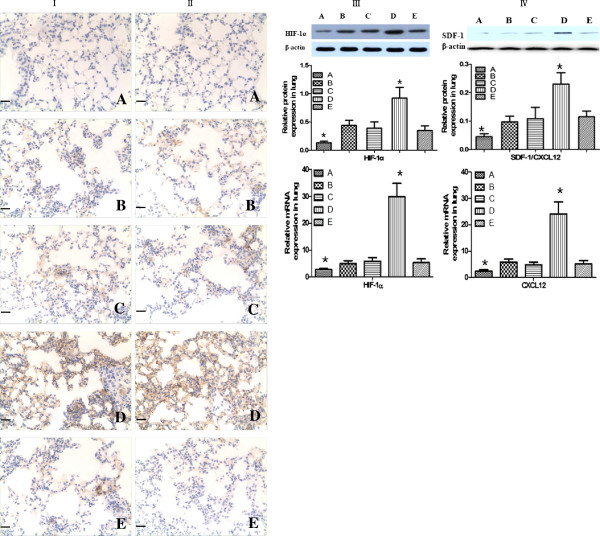
**HIF-1 and SDF-1 induce the remodeling of the lung after MSC transplantation. A)** Normal control group; **B)** COPD group; **C)** COPD + ^60^CO γ irradiation + MSCs transplantation; **D)** COPD + rAAV-SPA-TK injection + ^60^CO γ irradiation + MSC transplantation; **E)** COPD + AAV injection + ^60^CO γ irradiation + MSC transplantation. Analysis of HIF-1α **(I**, **III)** and SDF-1 **(II**, **IV)** expression in lung tissue by immunohistochemistry **(I**, **II)**, western blotting, and real-time PCR **(III**, **IV)**. The cells with yellow cytoplasm expressed HIF-1α and SDF-1. HIF-1α and SDF-1 were mainly expressed in airways and alveolar epithelia, and in the vascular endothelium and macrophages. No cells with yellow cytoplasm were found in group **A**. The mRNA and protein expression of HIF-1α and SDF-1 were increased in the other four groups, but there was no difference between groups **B**, **C**, and **E**. In group **D**, the expression of HIF-1α and SDF-1 was increased by several fold compared with that in groups **B**, **C**, and **E**. Scale bars = 100 μm. The results are expressed as the mean ± SD, n = 10 in each group.*P < 0.01 compared with the other four groups.

Cells expressing HIF-1α and SDF-1 were revealed by yellow staining of the cytoplasm. HIF-1α and SDF-1 were mainly expressed in airways and alveolar epithelia. Many cells with yellow-stained cytoplasm were found in all groups except for the normal control group, indicating increased expression of HIF-1α and SDF-1. However, there was no significant difference among COPD, the COPD + ^60^CO γ irradiation + MSC transplantation, and COPD + AAV injection + ^60^CO γ irradiation + MSC transplantation groups. Furthermore, HIF-1α and SDF-1 expression were significantly increased in the COPD + rAAV-SPA-TK injection + ^60^CO γ irradiation + MSC transplantation group compared with those in the other groups (Figure 4I, II). Moreover, the levels of HIF-1α and CXCL12 mRNAs and proteins were increased significantly in the COPD + rAAV-SPA-TK injection + ^60^CO γ irradiation + MSC transplantation group (Figure 4III, IV). These data suggest that rAAV-SPA-TK injection induces high expression of HIF-1α and SDF-1 that are possibly involved in the recruitment of MSCs to AT II niches and MSC differentiation in COPD.

### Increased IL-6, IL-8, and TNF-α levels in BALF of each COPD group

The levels of IL-6, IL-8, and TNF-α in BALF of groups B, C, D, and E were significantly increased compared with those in the control group. The expression of IL-8 and TNF-α showed no significant differences among groups B, C, D, and E, and the expression of IL-6 was significantly decreased in MSC groups compared with that in group B. These results indicate that AAV-SPA-TK/AAV injection and ^60^CO γ irradiation does not increase production of these proinflammatory factors in the rat lung (Table 2).

**Table 2:** Cytokines levels in BALF of each group (mean ± SD)

**Groups**	**n**	**IL-6 (pg/ml)**	**IL-8 (pg/ml)**	**TNF-α (pg/ml)**
A	10	212.93 ± 24.56*	74.37 ± 15.79*	14.22 ± 3.74*
B	10	826.62 ± 57.95*	281.54 ± 48.36	40.76 ± 5.21
C	10	593.04 ± 58.07	274.26 ± 52.81	37.54 ± 5.48
D	10	604.76 ± 48.84	293.6 ± 61.13	35.36 ± 5.66
E	10	621.37 ± 45.57	297.25 ± 40.04	36.28 ± 5.85

*P < 0.01 compared with the other four groups.

### SDF-1 is upregulated by HIF-1α under hypoxic conditions

**Figure 5:**
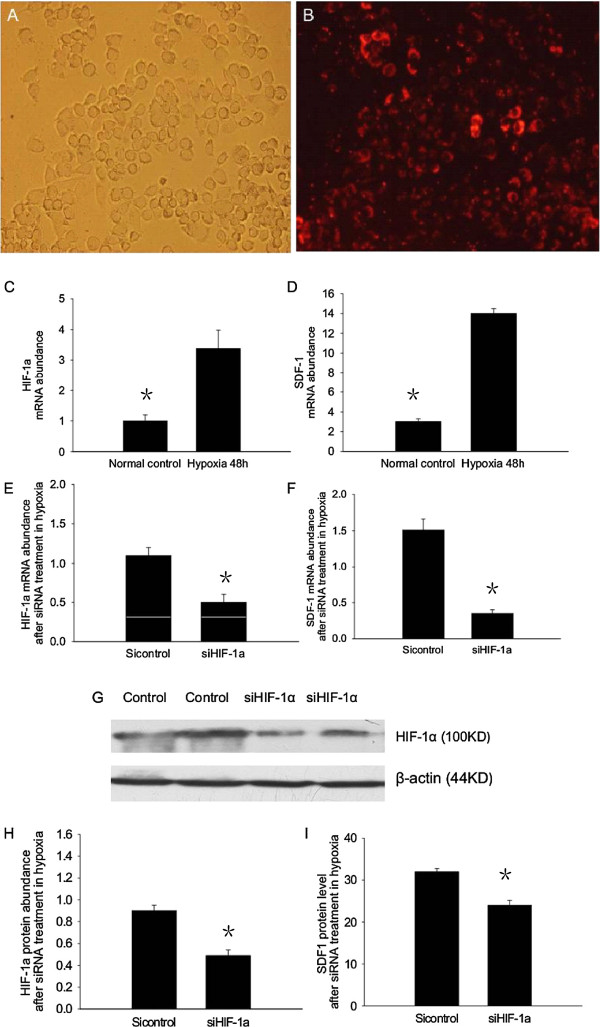
**SDF-1 is upregulated by HIF-1α under hypoxia.** First, we investigated changes in the expression of HIF-1α and SDF-1 under hypoxic conditions. The expression of HIF-1α and SDF-1 mRNAs increased significantly in A549 cells under hypoxic conditions compared with that under normoxic conditions **(C**, **D)**. Next, we investigated the effects of transfecting A549 cells with siRNA against HIF-1α. Control siRNA was labeled with red fluorescence. Using Lipofectamine™ 2000, an 80% siRNA transfection efficiency was achieved at approximately 6 h **(A**, **B)**. The expression of HIF-1α and SDF-1 mRNAs was decreased significantly in A549 cells transfected with siRNA against HIF-1α compared with that in A549 cells transfected with control siRNA **(E**, **F)**. Western blotting showed that the expression level of HIF-1α protein was also decreased significantly in A549 cells transfected with siRNA against HIF-1α **(G**, **H)**. ELISA results demonstrated that the level of SDF-1 was decreased significantly in the culture medium of A549 cells transfected with siRNA against HIF-1α **(I)**. The results are expressed as the mean ± SD, n = 10 in each group (*P < 0.05).

A549 cells were incubated under normoxic (21% oxygen) and hypoxic (1.5% oxygen) conditions for 48 h. The expression levels of HIF-1α and SDF-1 mRNAs were increased significantly in A549 cells under hypoxic conditions compared with those in normoxic conditions. This result suggests that hypoxia increases the expression of HIF-1α and SDF-1 mRNAs (Figure 5C,D). A549 cells were then transfected with siRNA against HIF-1α under hypoxic conditions. The transfection efficiency was 80% at approximately 6 h after transfection (Figure 5A,B). After siHIF-1α transfection of A549 cells with under hypoxic conditions, real-time PCR showed a decrease in HIF-1α mRNA expression and western blotting showed a decrease in HIF-1α protein expression (Figure 5E,G,H). The level of SDF-1 mRNA was also decreased significantly in A549 cells transfected with siHIF-1α compared with that in A549 cells transfected with control siRNA (*P* < 0.05) (Figure 5F). SDF-1 protein levels were also decreased significantly in the culture medium of A549 cells transfected with siHIF-1α as shown by ELISA (Figure 5I). These data suggest that hypoxia increases the expression of HIF-1α and SDF-1, and that SDF-1 expression is augmented by HIF-1α under hypoxic conditions.

## Discussion

Many studies have provided direct evidence that MSCs can potentially be used for the treatment of COPD [2-6]. Because the homing capacity of MSCs to the lung is low, we established a SPA suicide gene system to increase the rates of engraftment and differentiation of MSCs [7]. Our data suggested that transfection of the rAAV-SPA-TK vector increased the recruitment of MSCs, but collagen deposition by MSCs was also found to be increased during lung repair.

The airway epithelium is subjected to a lifetime of exposure to inhaled particles and pathogens, which may lead to the development of a variety of respiratory diseases such as COPD and cystic fibrosis [14]. Tissue injury and repair are ongoing processes in the lung because of exposure to these environmental insults [2-6,15].

Analyses of lung injury models have suggested that AT II cells may act as progenitor cells during lung injury [16,17], and they reside in their own niche [18]. However, in COPD, the available number of these cells for the repair and regeneration of damaged alveoli is limited because of excessive apoptosis in airway epithelial cells [19,20]. To enhance the repair capacity of the lung, two approaches have been taken into consideration. The first approach involves increasing the number of MSCs by, for example, exogenous MSC transplantation, and the second approach involves directly modifying the niche to be more amenable to repair. In this study, we investigated the simpler approach of MSC transplantation. However, the engraftment and differentiation rates of these cells were extremely inefficient even in the presence of lung injury, as shown by comparing the results of COPD and COPD + ^60^CO γ irradiation + MSCs transplantation groups. We therefore considered whether the engraftment and differentiation of exogenous MSCs was improved in the AT II cell niche.

In a previous study, we successfully established a HSV-TK/GCV killing system [21,22] by packaging the lung stem cell (AT II cell)-targeted virus, rAAV-SPA-TK, driven by the SPA promoter. Our results showed that more vacant AT II cell niches were obtained in COPD rats following rAAV-SPA-TK injection. In this study, we used rAAV-SPA-TK as a tool to achieve intact AT II cell niches to further study the *in vivo* recruitment and differentiation of transplanted MSCs. Obvious collagen fiber deposition was found in addition to the increased homing of exogenous MSCs. This finding suggested that rAAV-SPA-TK injection initiated secondary damage to AT II cells to produce many vacant niches, which increased the homing of MSCs and played an important role in their recruitment and differentiation. However, the observation that collagen fibers increased significantly around bronchi, vessels, and lung interstitial spaces in the rAAV-SPA-TK injection group indicated that MSC differentiation was more biased toward pulmonary fibrosis than the expected repair. The mechanism underlying this process is unclear. Recent studies have indicated that lung MSCs are triggered to differentiate into myofibroblasts by local factors. Accordingly, we believe that it is not the MSCs themselves (endogenous or exogenous MSCs) but the perturbation of diseased AT II cell niches that alter the potential migration or differentiation of MSCs.

The signaling pathways in the pulmonary niche, which are involved in the abnormal programming of MSCs, are unknown. Traditionally, HIF-1α has been recognized as a master regulator of O_2_ homeostasis and a key mediator of adaptive responses to tissue hypoxia. It plays a central and general role by signaling the existence of hypoxia to the transcriptional machinery in the nucleus of all cells. HIF-1α activates numerous target genes whose products are involved in angiogenesis and tissue remodeling [23,24]. Alveolar hypoxia has always existed in COPD because of hypoventilation or disturbances in pulmonary ventilation/perfusion matching [25]. Using a mouse model of orthotopic airway allograft transplantation, researchers have found that hypoxia promotes fibrogenesis *in vivo via* HIF-1α stimulation [26,27]. In our study, HIF-1α expression increased in all COPD rat model groups, and HIF-1α expression was significantly increased in the COPD + rAAV-SPA-TK injection + ^60^CO γ irradiation + MSC transplantation group compared with that in other groups. These data suggest that rAAV-SPA-TK injection induces high expression of HIF-1α by killing AT II cells, which possibly involves MSC differentiation in the AT II cell niche in COPD.

SDF-1 has been implicated as an important regulator of various stem cell functions including migration and differentiation [28]. Many studies have shown that the SDF-1/CXCR4 biological axis plays an important role in the pathogenesis of idiopathic pulmonary fibrosis [29]. Under hypoxia, the SDF-1/CXCR4 axis accelerates extracellular matrix deposition, resulting in the development and progression of idiopathic pulmonary fibrosis [30]. Our results showed that SDF-1 expression was significantly increased in the COPD + rAAV-SPA-TK injection + ^60^CO γ irradiation + MSC transplantation group compared with that in other groups. Therefore, increased expression of HIF-1α and SDF-1 may be involved in regulating the function and homing of MSCs, and increased collagen fiber deposition may result from high expression of HIF-1α and SDF-1. However, the relationship between HIF-1α and SDF-1 is still unclear.

We established an *in vitro* model of hypoxia using the AT II cell line A549. The expression of HIF-1α and SDF-1 increased under hypoxic conditions. When the cells were transfected with siHIF-1α, the expression of HIF-1α was knocked down at mRNA and protein levels, while SDF-1 was also decreased at mRNA and protein levels. These data suggest that SDF-1 expression is upregulated in AT II cells under hypoxia, and this process may be regulated by HIF-1α signaling.

## Conclusions

Vacating AT II cell niches by apoptosis increases the homing and recruitment of exogenous MSCs to the lungs of rats with COPD. These stem cell niches play a more important role in the lung than the MSCs themselves, and HIF-1α and SDF-1 signaling are involved in this process. In addition to the improvement of pathological changes in the lung, fibrosis is a serious problem presented by cell therapy for COPD. HIF-1α and SDF-1 are potentially promising therapeutic targets for such pathological processes. The existence of pulmonary fibrosis in COPD rat models following MSC transplantation is similar to combined pulmonary fibrosis and emphysema (CPFE). The histopathological coexistence of pulmonary fibrosis and emphysema first appeared in the literature in the 1970s [31]. Subsequently, additional clinical and pathological characteristics have been reported for CPFE, including its pathogenesis and treatment [32]. However, the molecular pathogenesis of CPFE is still unclear. The possible engraftment and differentiation of mesenchymal stem cells as a reason for the coexistence of pulmonary emphysema with pulmonary fibrosis is very exciting. Based on our findings and mounting clinical evidence, we hypothesize that abnormal repair of MSCs caused by primary and secondary damaged alveolar epithelial cell niches might play a role in the pathogenesis of CPFE. Our data may help to explain why only some smokers develop CPFE. Moreover, our suicide gene system against AT II cells or other factors may potentially be used to induce secondary damage. Further investigation of the role of endogenous lung stem cells (AT II cells) and their niche will provide additional insight into the mechanisms of lung development and repair after injury.

## Abbreviations

AT II: Alveolar epithelial type II; MSCs: Mesenchymal stem cells; HIF-1α: Hypoxia inducible factor-1α; SDF-1: Stromal cell-derived factor-1.

## Competing interests

The authors declare that they have no competing interests.

## Authors’ contributions

Conceived and designed the study: XMS, WGZ, and HLZ. Performed the animal experiments: WGZ, LH, and XMS. Performed the cell experiments: SSW, BZ, LM. Analyzed the data and prepared results: HLZ, XMS, and LH. Wrote the manuscript: HLZ and LH. Study supervised and coordinated: YJX, ZXZ, and JPZ. All authors read and approved the final manuscript.

## Supplementary Material

Additional file 1: Figure S1 Apoptosis of alveolar epithelial cells. TUNEL assays were performed on the rat lung. Nuclei were stained yellow in apoptotic lung cells. The numbers of these cells were significantly increased in COPD, COPD + rAAV-SPA-TK, and COPD + AAV groups compared with those in the control group. The highest number of apoptotic cells was in the COPD + rAAV-SPA-TK group. The percentage of TUNEL-positive cells was calculated by the ratio of TUNEL-positive cells to the total cell number in 10 fields at 400× magnification from each section. Scale bars = 100 μm. *P < 0.01 compared with the other three groups. Click here for file
