# Supplementary figures and images for: Regulation of transplanted mesenchymal stem cells by the lung progenitor niche in rats with chronic obstructive pulmonary disease
Source: Respir Res. 2014 Mar 25;15(1):33. doi: 10.1186/1465-9921-15-33 (PMC3987841; doi:10.1186/1465-9921-15-33)

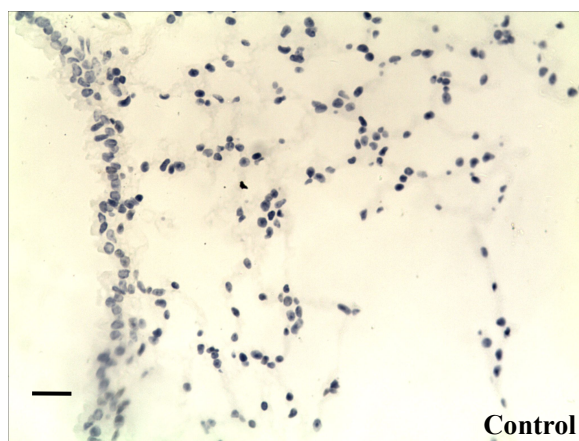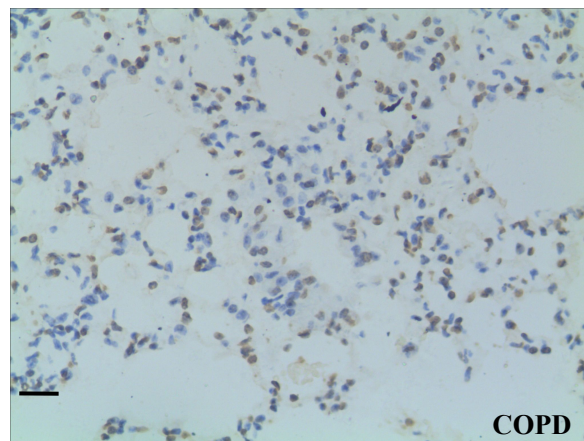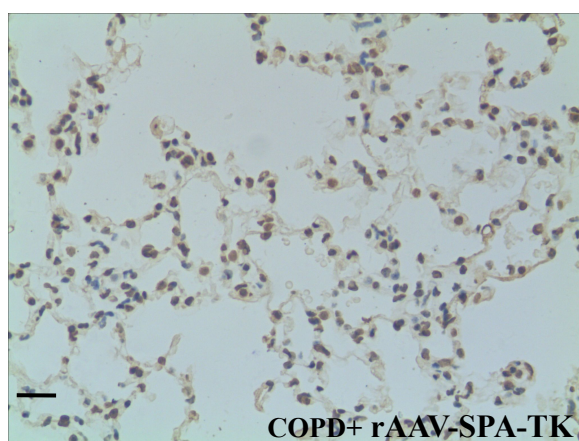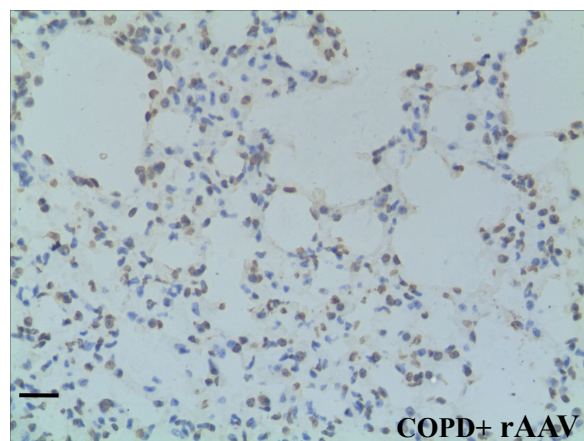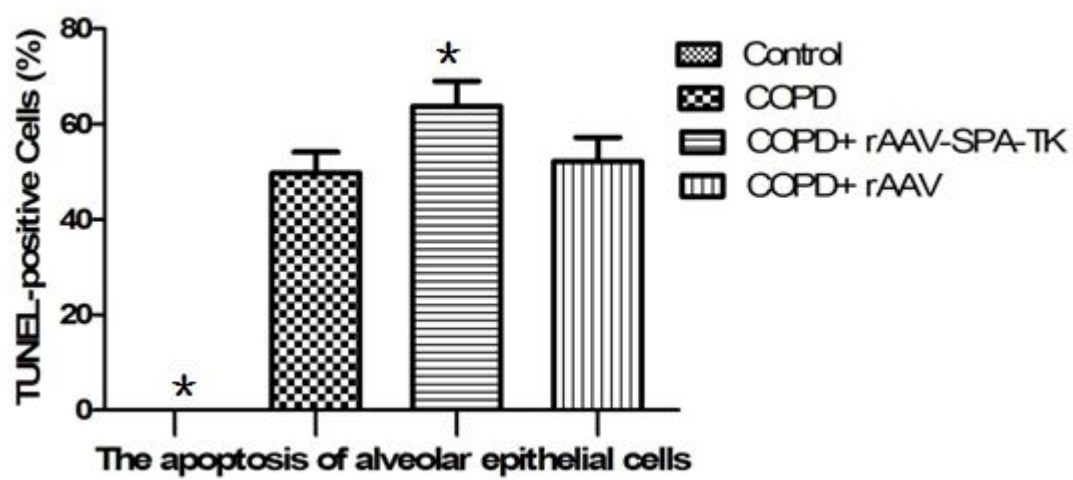

Supplement: Additional file 1: Figure S1 — Apoptosis of alveolar epithelial cells. TUNEL assays were performed on the rat lung. Nuclei were stained yellow in apoptotic lung cells. The numbers of these cells were significantly increased in COPD, COPD + rAAV-SPA-TK, and COPD + AAV groups compared with those in the control group. The highest number of apoptotic cells was in the COPD + rAAV-SPA-TK group. The percentage of TUNEL-positive cells was calculated by the ratio of TUNEL-positive cells to the total cell number in 10 fields at 400× magnification from each section. Scale bars = 100 μm. *P < 0.01 compared with the other three groups. [file 1465-9921-15-33-S1.pdf]
